# Stereo-Electroencephalography-Recorded Interictal Epileptiform Discharges: Activation Pattern and Its Relationship With Surgical Outcome

**DOI:** 10.7759/cureus.41337

**Published:** 2023-07-03

**Authors:** Cindy Luan, Jacob Miller, Caleb Sollars, Juan Peng, Jaysingh Singh

**Affiliations:** 1 Neurology, The Ohio State University Wexner Medical Center, Columbus, USA; 2 Biomedical Engineering, The Ohio State University College of Medicine, Columbus, USA; 3 Neurodiagnostic EEG Lab, The Ohio State University College of Medicine, Columbus, USA; 4 Biomedical Informatics, The Ohio State University College of Medicine, Columbus, USA

**Keywords:** irritative zone, epileptic seizure, intracranial eeg monitoring, interictal epileptiform discharges, stereo-encephalography

## Abstract

Background

Patients with drug-resistant epilepsy commonly undergo stereo-electroencephalography (SEEG) intracranial monitoring for surgical evaluation. Our current practice of defining the epileptogenic zone relies heavily on recognizing the seizure onset zone (SOZ), but the clinical significance of interictal epileptiform discharges (IEDs) is not well established.

Methodology

We retrospectively identified adult patients who underwent SEEG between January 2019 and May 2022. To study IED activation patterns, we classified IEDs as leading spikes (involved within the SOZ) and distant spikes (outside the SOZ). We calculated each patient’s total number of brain subregions generating distant spikes. We correlated them with epilepsy type, duration, and surgical outcome (Engel I: good outcome and Engel II-IV: poor outcome).

Results

A total of 22 patients were identified during the study period, and 16 underwent surgical intervention (ablation or resection) with one-year post-surgery follow-up. The most common IED morphology was a single spike or sharp followed by periodic spikes or sharps. We found that 87% (n = 19/22) of leading spikes were activated during the first 24 hours of SEEG monitoring, whereas no activation pattern was observed for distant spikes. We found that a higher number of subregions generating distant spikes were associated with poor surgical outcomes (p = 0.002). However, we did not find any significant association between the number of subregions generating distant spikes with epilepsy duration (p = 0.67), temporal or extratemporal-onset epilepsy (p = 0.58), or the presence of an MRI lesion (p = 0.62).

Conclusions

IEDs involved within the SOZ were found to be activated during the first 24 hours of SEEG monitoring, which could aid in recognizing the pathological spikes and targeted mapping of the irritative zone. We also observed that a higher number of brain subregions generating IEDs outside the SOZ were associated with poor surgical outcomes, but this observation needs to be further studied with larger sample size prospective studies.

## Introduction

Epilepsy is a chronic neurologic disorder that affects over 3.4 million people nationwide. One-third of patients with epilepsy have seizures refractory to pharmacotherapy and deemed drug-resistant epilepsy (DRE) [1]. Patients with DRE have increased risks of sudden unexpected death in epilepsy, physical injuries, psychosocial dysfunction, and reduced quality of life. Frequently, patients with DRE undergo stereo-electroencephalography (SEEG) intracranial recording to define the epileptogenic zone (EOZ) [2,3], defined as the minimum amount of cortex requiring resection to achieve seizure freedom. The relationship between brain regions generating interictal epileptiform discharges (IEDs) (referred to as the irritative zone (IZ)) and areas involved in the initiation of the seizure (referred to as the seizure onset zone (SOZ)) is quite complex. It is generally believed that the IZ is larger than the SOZ [4]. Jasper and Penfield have shown that interictal discharges can be seen in areas other than the SOZ and tissue distant from the lesional site [5]. Our current practice uses the SOZ as a proxy marker to define the EOZ [6], which has not translated into better surgical outcomes. Only 50% of patients undergoing surgery achieve seizure freedom, highlighting the need for more markers for defining the EOZ [7].

The relationship between IEDs, SOZ, and surgical outcomes remains an important research focus. In this study, we classified IEDs involved in the SOZ as leading spikes and those outside the SOZ as distant spikes to study their activation features during SEEG monitoring. We also studied morphology, the number of brain subregions generating distant spikes, their relationship with epilepsy types, epilepsy duration, epileptogenic lesions on brain imaging, and surgical outcomes.

## Materials and methods

Study subjects

We retrospectively identified consecutive adult patients (>18 years of age) with DRE who underwent SEEG intracranial monitoring to localize the seizure focus before focal resective surgery between January 2019 and May 2022. Patients who underwent subdural intracranial EEG monitoring were excluded. The Institutional Review Board approved this study at the Ohio State University Wexner Medical Center.

Clinical data were collected from the electronic medical record, including baseline demographic data (age and gender); type of epilepsy and seizure history; past medical history; findings of brain imaging which included MRI brain imaging, functional MRI of the brain, positron emission tomography (PET) scan of the brain, and single-photon emission computerized tomography of the brain; neuro-psych cognitive findings; scalp EEG findings about epilepsy surgery; pathology report; and surgical outcomes.

JS and CL reviewed SEEG intracranial data. The average length of stay following SEEG implantation was 8.2 days. We classified the morphology of IEDs as single sharp or spike, periodic sharp or spikes, polyspikes, and then IEDs involved in the SOZ as leading spikes and those outside the SOZ as distant spikes. We calculated the number of brain subregions that generated IEDs. Independent epileptic spikes were recognized as a single subregion generating that type of IED. Spikes that had a broad field and were seen over the multiple SEEG contacts counted toward the subregion that generated spikes with maximum negativity. Similarly, local spike activity has a tendency for regions to co-activate during spike discharges with millisecond-scale latencies and are classified as propagated spikes. These IEDs were counted toward the subregion generating the spikes with maximum negativity that led to a propagated spike.

Surgical outcomes were classified as per Engel outcomes, with a minimum of one-year post-surgery follow-up. Engel I outcome was deemed good surgical outcomes, whereas Engel II-IV outcomes were deemed poor surgical outcomes.

Statistical analysis

Univariate logistic regression models were used to examine the associations with having good Engel outcomes. Odds ratios (ORs) and 95% confidence intervals (CIs) were reported. For counts less than 5, Fisher exact test was performed. Wilcoxon rank-sum test was used to examine the difference in the number of IED pockets and dominant spikes among categorical variable levels. A Spearman correlation test was performed on the relationship between epilepsy duration and the number of IED pockets and dominant spikes. A two-sided significance level of 0.05 was used for all tests. All analyses were done in SAS version 9.2 (SAS Institute, Cary, NC, USA).

## Results

A total of 22 adult patients were identified who underwent SEEG monitoring during the study period, of which 68% (n = 15) were temporal lobe epilepsy cases. The most common etiology for epilepsy was cryptogenic (45.4%), lesion on MRI (61.9%), and abnormal PET (63.6%). Sixteen patients underwent surgical resection or ablation (72%, n = 16/22); the others were deemed unsuitable for surgery and underwent neuromodulation with deep brain stimulation or responsive nerve stimulation. Table 1 provides a summary of patient characteristics.

**Table 1:** Summary of patient characteristics. IEDs = interictal epileptiform discharges; PET = positron emission tomography

Characteristics	Entire sample, mean ± SD (median, range) or N (%)
Age	39.00 ± 12.66 (22, 36.5–66 years)
Sex	
Female	5 (22.73%)
Male	17 (77.27%)
Number of IED pockets	5.41 ± 1.87 (3, 5–8)
Number of subregions generating distant spikes	1.09 ± 1.02 (0, 1–4)
Number of electrodes	10.64 ± 2.36 (6, 10.5–15)
Etiology	
Cryptogenic	10 (45.45%)
Mesial temporal lobe sclerosis	5 (22.73%)
Tumor	2 (9.09%)
Meningitis/Encephalitis	2 (9.09%)
Traumatic brain injury	3 (13.64%)
Seizure type	
Focal to a bilateral tonic-clonic seizure	6 (27.27%)
Focal aware or focal with impaired awareness	16 (72.73%)
MRI brain	
Negative	8 (38.10%)
Presence of epileptogenic Lesion	13 (61.90%)
PET	
Normal	8 (36.36%)
Other	14 (63.64%)
Epilepsy type	
Extratemporal	7 (31.82%)
Temporal	15 (68.18%)
Surgery (resection or ablation)	16 (72%)

A total of 119 IED pockets were recorded, and a single spike or sharp wave was found, followed by periodic spikes or sharps as the most common morphology for leading and distant spikes. Taking the timing of the IED pocket, we found 86% of leading spikes were activated within the first 24 hours of SEEG monitoring, whereas no specific pattern was seen for distant spikes.

We found that a higher number of subregions generating distant spikes were correlated with poor surgical outcomes (n = 5/16, mean = 6, p = 0.002). We did not observe any significant association between the number of subregions generating distant spikes with epilepsy duration (p = 0.67), temporal or extratemporal-onset epilepsy (p = 0.58), or the presence of an MRI lesion (p = 0.62). A summary of the analysis is shown in Table 2.

**Table 2:** Summary of distant spikes analysis.

Analysis variable: Number of subregions generating distant spikes							
	N (16)	Mean	Standard deviation	Minimum	Median	Maximum	P-value
Engel outcomes							
Poor	5	6.00	0.71	5.00	6.00	7.00	0.0022
Good	11	2.73	1.10	1.00	3.00	5.00	
Epilepsy onset							
Extratemporal	3	3.00	1.00	2.00	3.00	4.00	0.58
Temporal	13	3.92	1.98	1.00	3.00	7.00	
Presence of MRI epileptogenic lesion							
Negative	6	4.17	2.32	1.00	4.50	7.00	0.62
Epileptogenic lesion	10	3.50	1.58	2.00	3.00	6.00	

## Discussion

In this retrospective study of 22 adult patients who underwent SEEG monitoring, 119 IED pockets were recorded and further classified as leading spikes (IEDs involved in the SOZ) and distant spikes (IEDs outside the SOZ). We found two important observations in our cohort: (1) leading spikes were activated during the first 24 hours of SEEG monitoring, and (2) a higher number of brain subregions generating distant spikes were correlated with poor surgical outcomes.

IEDs represent summated membrane events from hypersynchronous neuronal activity. Earlier work from Jasper et al. introduced this critical concept that not all spikes have the same pathological value. They reported that the spikes from the EOZ were sharper and associated with structural lesions [8]. Similarly, Serafini et al. observed subdural recorded IEDs exhibited variable spatial organization where spikes outside the EOZ (green spikes) showed a relative increase of the slow wave compared to IEDs within the EOZ (red spikes) [9]. Bartolomei et al. studied 31 patients who underwent SEEG monitoring and explored 539 brain regions to investigate the relationship between the spiking frequency and the SOZ. They reported good concordance between spiking frequency and the SOZ in the focal cortical dysplasia (FCD) group (75%) versus the non-FCD group (35%) [10]. Thomas et al. recently analyzed the spike features (rate, morphology, propagation, and energy) on stereo-EEG segments from 83 operated patients [11]. They found the rate of spikes with preceding gamma activity in wakefulness performed better for the surgical outcome than the SOZ and the ripple rate. They also reported that the channels with a spike gamma rate exceeding 1.9/minute had an 80% probability of being in the EOZ. Our finding of early activation of leading spikes during SEEG monitoring could be explained by a higher proportion of patients with temporal lobe epilepsy where we commonly observe early activation of IEDs. This finding can guide the targeted mapping of the IZ using these advanced features and signal analysis, thereby understanding the relationship of the IZ with the EOZ.

Consistent with the literature, we found more brain subregions generating IEDs than being involved at seizure onset, confirming that the IZ is more extended than the EOZ and can be separated into a primary IZ, which includes the SOZ, and a secondary IZ, which roughly corresponds to areas of seizure propagation [12,13]. The spatial distribution of the IEDs can be highly variable, as observed by Janca et al. in neocortical seizures with multiple subregions generating IEDs, each with specific IED propagation trajectories and differing in the extent of the IED activity generated [14]. They also noted that the global activity of IEDs is influenced by circadian fluctuations of spatiotemporal properties in subregions, and the most active subregion co-localizes with the SOZ. Similarly, Paolicchi et al. classified IEDs into active spikes (continuous or bursting spiking patterns) and distant spikes (sporadic, infrequent) and demonstrated that resection of regions generating active spikes, but not of a distant spike, correlated with a good surgical outcome [15]. In our study, the higher number of subregions generating distant spikes (IEDs outside the SOZ) being associated with poor surgical outcomes can be explained by a higher proportion of extratemporal lobe epilepsy cases in our cohort, where we commonly observe more extended IZ compared to temporal lobe epilepsy. However, the number of patients who underwent surgery in our cohort is small, and this finding needs to be further studied with a larger sample size. We also did not analyze any associated features with distant spikes, such as the presence of high-frequency oscillations or preceding gamma activity. More prospective studies are needed to evaluate the clinical significance of IEDs outside the SOZ.

Our study has several limitations, including its retrospective nature and the limited sample size. Second, SEEG electrode implantation is determined primarily by the suspected EOZ, highlighting the limited spatial sampling and underestimating the real burden of IEDs. Lastly, most of our cases were of temporal lobe epilepsy and a limited range of pathologies. Nevertheless, the results of this study suggest the need to closely analyze the IEDs in patients undergoing SEEG monitoring to define the epileptogenic zone, particularly in extratemporal lobe epilepsy for resective surgery.

## Conclusions

IEDs involved within the SOZ were found to be activated during the first 24 hours of SEEG monitoring, which could aid in recognizing the pathological spikes and targeted mapping of the IZ. We need more prospective studies to study the significance of IEDs generated outside the SOZ.
